# pH-Induced Changes in the SERS Spectrum of Thiophenol at Gold Electrodes during Cyclic Voltammetry

**DOI:** 10.1021/acs.jpcc.2c00416

**Published:** 2022-04-20

**Authors:** Jorn D. Steen, Anouk Volker, Daniël R. Duijnstee, Andy S. Sardjan, Wesley R. Browne

**Affiliations:** Molecular Inorganic Chemistry, Stratingh Institute for Chemistry, Faculty of Science and Engineering, University of Groningen, Nijenborgh 4, Groningen 9747AG, Netherlands

## Abstract

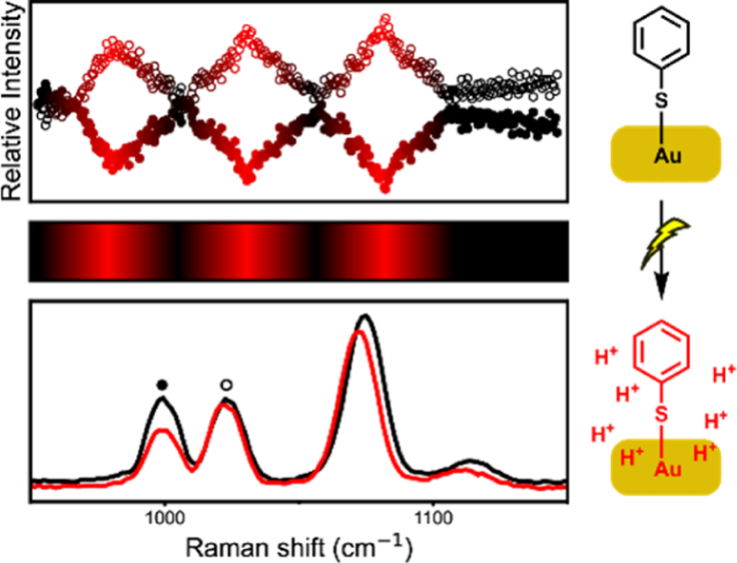

Thiophenol is a model compound used in the study of self-assembly of arylthiols on gold surfaces. In particular, changes in the surface-enhanced Raman scattering (SERS) spectra of these self-assembled monolayers (SAMs) with a change of conditions have been ascribed to, for example, differences in orientation with respect to the surface, protonation state, and electrode potential. Here, we show that potential-induced changes in the SERS spectra of SAMs of thiophenol on electrochemically roughened gold surfaces can be due to local pH changes at the electrode. The changes observed during the potential step and cyclic voltammetry experiments are identical to those induced by acid–base switching experiments in a protic solvent. The data indicate that the potential-dependent spectral changes, assigned earlier to changes in molecular orientation with respect to the surface, can be ascribed to changes in the pH locally at the electrode. The pH at the electrode can change as much as several pH units during electrochemical measurements that reach positive potentials where oxidation of adventitious water can occur. Furthermore, once perturbed by applying positive potentials, the pH at the electrode takes considerable time to recover to that of the bulk solution. It is noted that the changes in pH even during cyclic voltammetry in organic solvents can be equivalent to the addition of strong acids, such as CF_3_SO_3_H, and such effects should be considered in the study of the redox chemistry of pH-sensitive redox systems and potential-dependent SERS in particular.

## Introduction

Self-assembled monolayers (SAMs) of thiols on gold surfaces have played a central role in surface science since the 1980s with the seminal studies on gold surfaces by Nuzzo and Allara^1^ and Bain and Whitesides.^2^ The characterization of these SAMs, for example, the packing density,^3^ mobility, and nature of the bonding between the thiols and the gold surface, is ongoing, in particular efforts toward characterization of binding motifs on gold surfaces.^4−6^ The adsorption of thiophenol and its analogues is the focus of many theoretical and experimental studies.^4^ Properties of most interest are the nature of the bonding interaction between gold and sulfur, distinguishing between physisorption and chemisorption,^7−9^ the stability and prevalence of various binding modes,^5,8,10^ and the dependence of thiophenol self-assembly on conditions (e.g., pH, electrode potential, and temperature).^11^

The details of binding of thiols to gold are still under debate—in particular, the protonation state of the thiol—and is complicated by the dependence of binding and protonation states on solvent conditions as well as the nature of the alkyl/aryl group. Computational studies on atomically flat surfaces indicate that thiols bind primarily to gold together with an adatom,^8,12−14^ which is an atom of gold present on top of the surface due to the strong Au–S bond. For short chain alkanethiols, the relatively low energy of intermolecular interactions likely results in strengthening of Au–S bonds with respect to Au–Au bonds, effectively increasing the Au adatom mobility.^8^ While the adsorption energy of thiophenol on gold is not known, Gibbs free energies of adsorption have been reported for thiophenol on mercury^15^ and platinum,^16^ and theoretical calculations and comparisons with other thiols have led to an estimated energy of 167.15 kJ mol^–1^ for the Au–S bond, a relatively large value indicating strong chemisorption.^17^ On roughened gold surfaces, on the other hand, multiple adsorption sites are available, and at room temperature the binding geometry is subject to thermal fluctuations.^18^

**Scheme 1:**
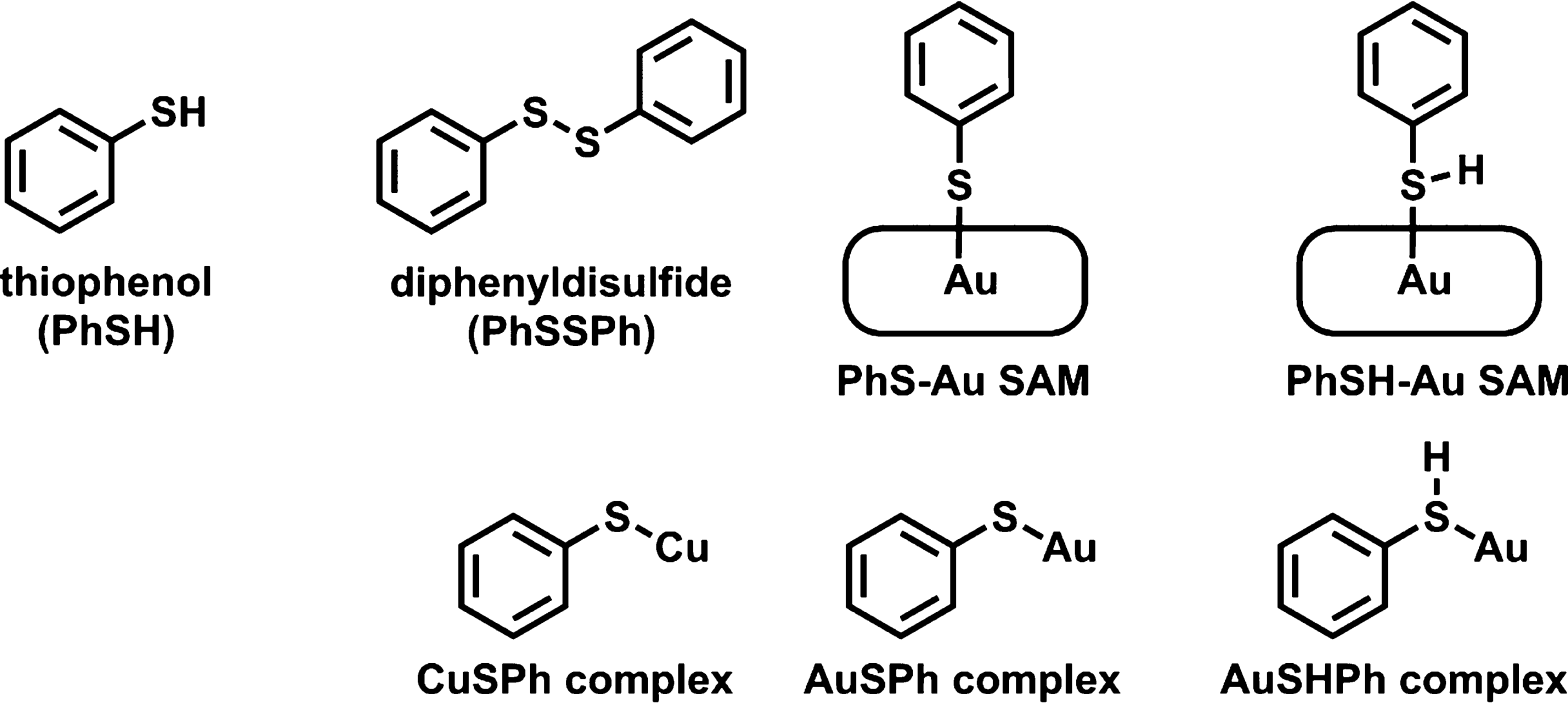
Compounds and Binding Modes Discussed in This Work

Similarly, the protonation state of the thiol is not necessarily clear. Based on density functional theory (DFT) calculations, it was concluded by Guesmi et al. that the length of the alkane in alkyl thiols has an influence on whether the thiol is protonated or not, more specifically that longer-chain alkanethiols favor retention of the S–H bond upon adsorption.^8^ Recently, Inkpen et al.^7^ used electronic conductance in single-molecule junctions of various dithiol-substituted compounds to determine the nature of the Au–S bond in SAMs prepared by solution deposition. They noted that measurements under scanning tunneling microscopy break junction conditions and calculations were not of an equilibrium state but nevertheless provide information on the bonding at equilibrium (i.e., chemisorption or physisorption) and concluded that the hydrogen/proton is not lost upon self-assembly onto gold during solution deposition from 1,2,4-trichlorobenzene at room temperature. For arylthiols, however, such solvation effects are less likely to play a role and they are intrinsically more acidic than alkyl thiols. Indeed, when immersed in proton-accepting solvents, the adsorbed thiophenol can easily lose its proton to the surrounding solvent (i.e., solvent leveling, especially when solvent contains adventitious water, for example, in acetonitrile or ethanol), resulting in the formation of a **PhS-Au** species (Scheme 1).^19^

Among the wide range of techniques applied to date to study alkyl-/arylthiol SAMs, vibrational spectroscopy is especially informative as it enables (changes in) the molecular structure of adsorbed thiols to be probed. The surface plasmon of rough gold surfaces provides enhancement of the Raman scattering intensity when excitation is resonant with the plasmon absorption band, referred to as surface enhanced Raman scattering (SERS),^20,21^ overcoming the limitations in signal intensity arising from the low number density of molecules in the confocal volume.^22−24^ For instance, the dependence of the adsorption of thiophenol on gold on various conditions (e.g., pH) was studied by Tripathi et al. using SERS spectroscopy.^11^ The binding of thiophenol to Au was found to be pH-dependent: at low pH, the rate of SAM formation is limited by the rate of physisorption, while at high pH the rate-limiting step is chemisorption due to a majority of molecules being in the (deprotonated) thiophenolate state already in solution (**PhS-Au**, Scheme 1).^11^

The SERS spectra of **PhS-Au** SAMs are distinctly different from those of neat **PhSH**, in terms of the Raman shifts of specific bands. These differences were attributed by Szafranski et al. to the orientation of the aromatic ring with respect to the surface.^25^ Carron and Hurley drew a similar conclusion and related the difference in the ratio of two Raman bands of thiophenol adsorbed to gold, silver, and copper to different axial and azimuthal angles of the molecule with respect to the surface.^26^

The effect of electrode potential on thiophenol SAMs is also of interest. Holze reported an increase in SERS intensity and a concomitant shift to a lower wavenumber of the Au–S bond (265 cm^–1^) at more positive potentials in HClO_4_ (aq).^27^ In contrast, in KClO_4_ (aq), the same Raman band shifts to higher wavenumbers upon cycling to positive potentials, which was rationalized by the presence of deprotonated thiol species (**PhS-Au**) at higher pH, which binds more strongly. Although not commented on specifically, the SERS data indicate a change in the ratio of the bands at 1000 and 1025 cm^–1^ during cyclic voltammetry also. The spectral changes during cyclic voltammetry described by Holze^27^ were noted by Yokota et al. also in studies with thiophenol as a standard for tip-enhanced Raman scattering (TERS) spectroelectrochemistry.^28^ Changes in relative intensities and peak positions of Raman bands around 1000 cm^–1^ were observed at potentials close to that of oxidative desorption of **PhS-Au** SAM (0.7 V vs Au, i.e., 1.15 V vs Ag/AgCl). More precisely, the relative intensity of the band at 1025 cm^–1^ increased versus that at 1000 cm^–1^ as the electrode potential was cycled from 0.05 to 1.05 V (vs Ag/AgCl). Hong et al., however, noted a variation in intensity of the bands at 1000 and 1025 cm^–1^ as a surface was scanned using TERS spectroscopy;^29^ hence, although the intensity of these bands are likely to be sensitive to structure and charge transfer interactions between the molecules and the substrate, it remains unclear as to the specific changes in conditions that are responsible.

Here, we show that the changes in the SERS spectrum of thiophenol SAMs on gold during cyclic voltammetry are identical to those induced by a deliberate increase of proton concentration through addition of strong acid, and we ascribe this to a significant increase in proton concentration at the electrode at positive potentials where oxidation of water occurs. The reversible change in relative intensities of the characteristic bands at 1000 and 1025 cm^–1^, noted in the earlier data reported by Holze^27^ and by Yokota et al.,^28^ and the associated change in the molecular structure, conformation, or orientation, therefore show the relation with a substantial change in local pH at the electrode, where the self-assembled monolayers are located. The effect of pH on the relative intensity of the bands also enables real-time monitoring of the local pH at electrodes during potential step experiments and cyclic voltammetry, and we show that in organic solvents the change in local pH can be equivalent to the addition of strong acids such as trifluoromethanesulfonic (triflic) acid.

## Methods

Reagents and solvents were obtained from Sigma-Aldrich and used as received unless stated otherwise. Copper^26^ and gold^30,31^ complexes of thiophenol were prepared using reported procedures. The gold complex (**AuSPh**) was prepared by addition of excess thiophenol to a solution of HAuCl_4_·*x*H_2_O in a water/methanol mixture. The copper complex (**CuSPh**) was prepared similarly from CuCl_2_. The Raman^26,32^ and Fourier transform infrared (FTIR)^33^ spectra of the compounds obtained were consistent with the spectra reported earlier (Figures S1 and S2).

Gold beads were prepared by melting gold wire in butane flame. The beads were roughened electrochemically using a literature procedure^34^ with a CH Instruments 760C bipotentiostat and verified optically as a darkening of the surface of the gold electrode (Figure S3). SAMs formed upon immersion in ethanol or acetonitrile containing thiophenol (0.1 M). The gold colloid suspension in water was prepared according to the citrate method.^35^ Addition of thiophenol (1 μL) to 2 mL of aqueous Au colloid in a quartz cuvette with gentle mixing was followed by addition of conc. aqueous H_2_SO_4_ to bring the pH to ca. 0.5, and a Raman spectrum was recorded. The procedure was repeated, except that conc. aqueous KOH was added to bring the solution to a pH of ca. 13.

Raman spectra were recorded with excitation at 785 nm using an Olympus BX51 microscope equipped with a fiber-coupled laser (BT785, ONDAX) and a fiber-coupled Shamrock163i spectrograph and an iVac-DLL CCD camera and a 235 line/mm grating with 750 nm blaze. The power at the sample was varied from 1 to 300 mW and was typically 2–5 mW. Heating of solid samples was carried out in a TG84 instrument (Mettler Toledo) with optical access for Raman spectral measurements at 785 nm. Electrode potential was controlled with either a CHI760c or CHI604E potentiostat, a platinum counter electrode, and an SCE or Ag/AgCl reference electrode.

DFT geometry optimizations and frequency calculations of the Au_4_-thiophenolato clusters were performed with ORCA 5.0.3,^36,37^ using the default optimization algorithm. First, the gold clusters were optimized at the B3LYP/def2-SVP level,^38,39^ using the default def2-SVP effective core potentials for the Au atoms, with the def2/J auxiliary basis set,^40^ as well as electron smearing at 5000 K to aid SCF convergence. Next, the thiophenolato unit (-SPh or -SHPh) was added, and the molecular geometry was optimized at the aforementioned level while keeping the gold atoms frozen. Finally, the whole system was optimized at the B3LYP/ZORA-def2-TZVP level with a SARC/J auxiliary basis set^40,41^ and with a conductor-like polarizable continuum model^42^ of acetonitrile. See the Supporting Information for further details.

## Results and Discussion

The SERS spectra of thiophenol SAMs on roughened beads in air and immersed in a solvent (water, ethanol, or acetonitrile) are equivalent and consistent with those reported earlier (Figure S4 and Table S1).^19,26,27^ The spectra differ substantially from the non-resonant Raman spectra of thiophenol neat and in solution. In particular, the bands at 1585 and 1092 cm^–1^ in the spectrum of neat thiophenol shift, respectively, to 1575 and 1073 cm^–1^ in the surface-enhanced spectra, which is consistent with a change in the structure (coordination of the sulfur to gold). Comparison of SERS spectra of a number of **PhS-Au** SAMs, prepared from various stock solutions and using different roughened gold beads (Figure S4), reveals a variation in the relative intensities of the bands at 1000 and 1025 cm^–1^ as noted earlier by Yokota et al.,^28^ who attributed the differences to structure and charge transfer from the adsorbed thiophenol to the gold surface; however, a correlation could not be drawn between the relative intensities of these bands and the conditions used to prepare the SAMs. These observations prompted us to examine first the effect of coordination of thiophenol to copper and gold on its Raman spectrum and then establish a correlation between conditions and spectral changes in the SERS spectra of thiophenol SAMs on gold.

The complexes **AuSPh**(30,31) and **CuSPh** (Scheme 1),^26^ prepared according to literature procedures, were characterized by Raman spectroscopy (Figure S1). The Raman spectrum of the **CuSPh** complex obtained is essentially identical to that of **AuSPh**, and both show substantial differences to the spectrum of **PhSSPh**, not least the lack of the S–S stretch at 544 cm^–1^ (Figure S1).^25,26^ Both Raman spectra are in agreement with those reported for **CuSPh**, **AgSPh**, and **AuSPh** by Carron and Hurley^26^ and demonstrate how small a difference the nature of the metal thiophenolate has on its vibrational spectrum despite the difference in polarization of the Cu–S and Au–S bond predicted for thiolate complexes.^43^

**Figure 1:**
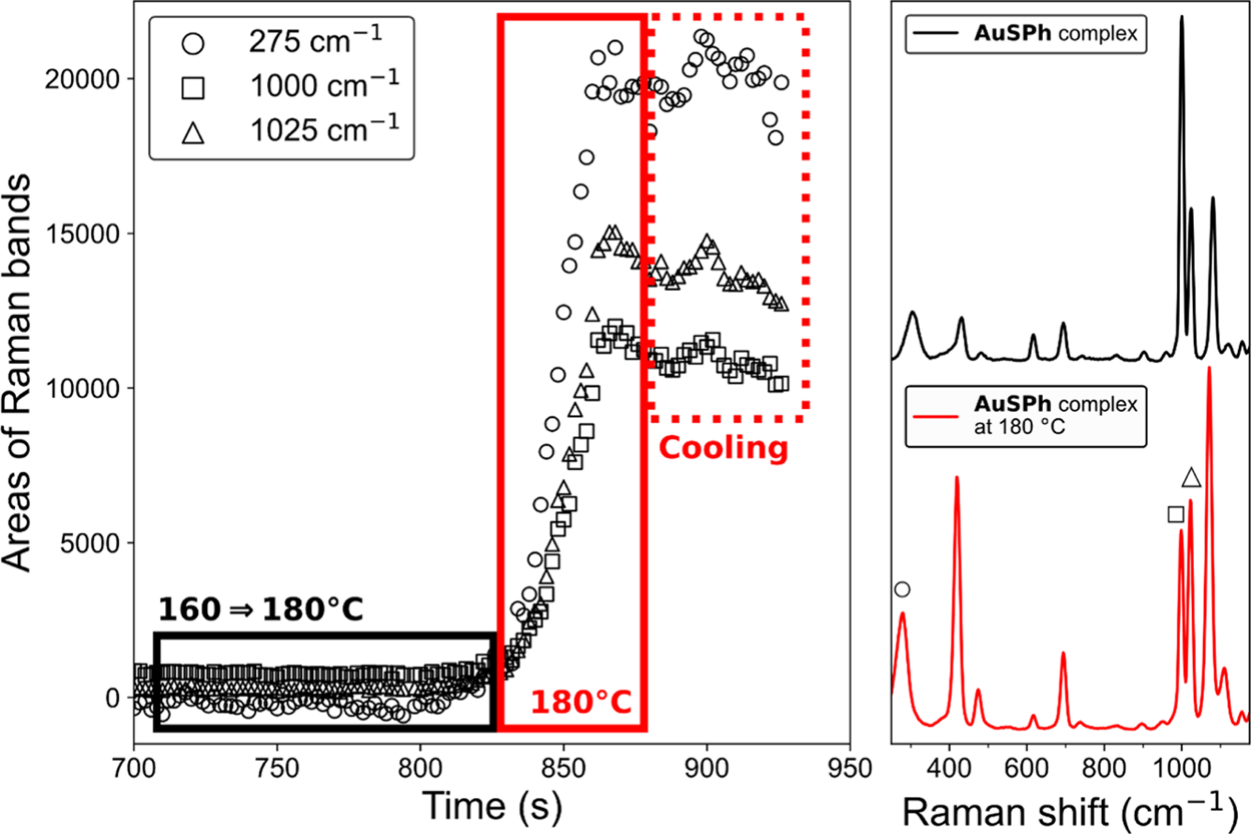
(Left) Intensity of several Raman bands of the **AuSPh** complex over time during heating in a microscope heating stage. The temperature increased monotonically from 160 to 180 °C between 700 and 825 s (black box) and was held at 180 °C until 875 s (solid red box) and thereafter allowed to cool rapidly (dashed red box). (Right) Raman spectra (λ_exc_ 785 nm) of the **AuSPh** complex before (black) and after (red) heating to 180 °C. The spectra are normalized and offset for clarity.

The Raman spectrum of **AuSPh** was observed to change over time during spectral acquisition with an increase in overall spectral intensity and a change in relative band intensity and position. Indeed, over a relatively short time (ca. 4 s), the Raman spectrum changed to match that of the SERS spectrum of **PhS-Au** SAMs (Figure S5). These changes were not observed when the laser power was reduced from 30 to 1 mW at the sample even with prolonged exposure times. It was suspected that in situ thermal reduction of the complexes to form thiophenol-coated Au nanoparticles was responsible for these changes. Indeed, Demessence et al. have reported such reduction of a **(Au-SPh)**_***n***_ coordination polymer under calcination conditions combining high pressure and high temperature.^32^ Raman spectra of a solid sample of **AuSPh**, recorded with sufficiently low laser power to avoid sample heating, before, during, and after heating in a microscope heating stage showed a substantial increase in spectral intensity and a change in the position and relative intensity of the bands upon reaching 180 °C, eventually yielding a spectrum that matches the SERS spectrum of thiophenol on gold also (Figure 1). The attenuated total reflection (ATR)-FTIR spectrum of the thermally treated sample was, however, essentially identical to that of the initial compound, apart from a new unassigned band at 1730 cm^–1^ (Figure S6), indicating that the bulk sample was only slightly affected by heating and that the SERS enhancement due to reduction of a minor amount of the gold present to form nanoparticles was sufficiently strong to overwhelm the Raman scattering of the remaining bulk material. It is of note that, before heating, the ratio of the Raman bands at 1000 and 1025 cm^–1^ was consistent with SERS spectra obtained under basic conditions, while after heating induced changes to the sample, the ratio resembled those under acidic conditions (vide infra).

The protonation state of thiophenol in solution is readily controlled by addition of a base. For neat thiophenol, the band at 2575 cm^–1^ is assigned to the S–H stretching mode.^25,27,44^ Addition of base (triethylamine, Et_3_N) to a 0.5 M solution of thiophenol in CH_3_CN results in a decrease in intensity of this band consistent with partial deprotonation (Figure S7). Further significant differences are the appearance of new bands at 966 and 1170 cm^–1^ and the shift of the bands at 1095 and 1585 cm^–1^ to 1083 and 1575 cm^–1^, respectively (Table S1). The shift to lower wavenumber is consistent with deprotonation of thiophenol and moves the bands closer to their position in spectra of SAMs of thiophenol on gold, that is, at 1075 and 1575 cm^–1^ (Figure S4).

### pH Dependence of Thiophenol SAMs

**Figure 2:**
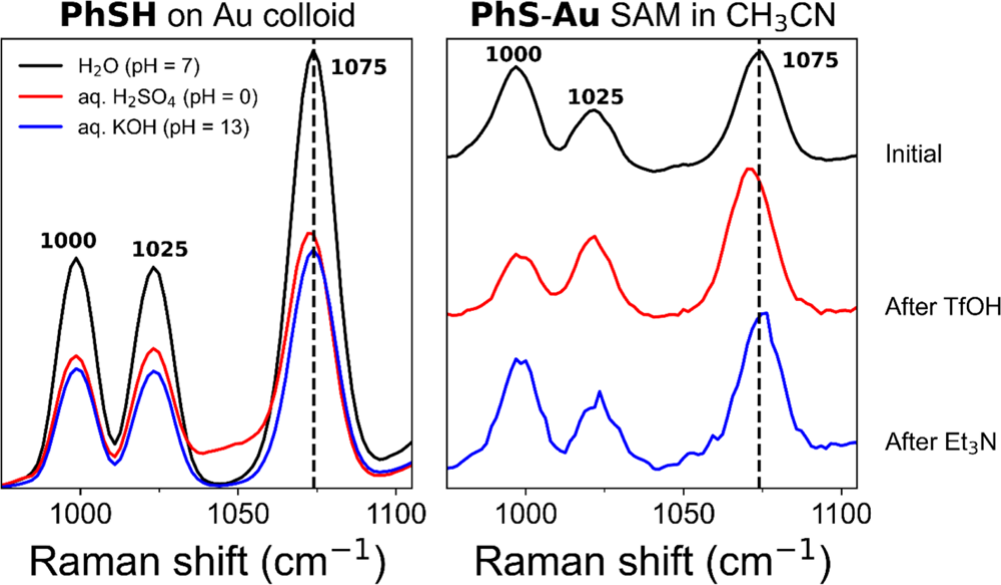
(Left) SERS spectra (λ_exc_ 785 nm) of **PhSH** on aggregated gold colloid in H_2_O (black) at pH = 0 (red) and pH = 13 (blue). (Right) SERS spectra (λ_exc_ 785 nm) of **PhS-Au** on a roughened gold bead in CH_3_CN before (black), after addition of TfOH (red), and after subsequent addition of Et_3_N (blue). The spectra are normalized and offset for clarity. For full spectral range, see Figures S8 (colloids) and Figures S8 and S9 (SAM on gold beads).

SERS spectra of **PhS-Au** SAMs show several subtle but notable changes as pH is varied: variation in the ratio of intensities of the Raman bands at 1000 and 1025 cm^–1^ and changes in the Raman shift of the bands at 1075 and 1575 cm^–1^ (Figure 2, and corresponding full spectra in Figures S8 and S9). The change in the ratio of intensities of the two bands is seen to a greater extent when induced by addition of CF_3_SO_3_H (TfOH) or triethylamine (Et_3_N) to the acetonitrile in which **PhS-Au** SAMs on roughened gold beads are immersed. The ratio *I*_1025_/*I*_1000_ increases upon addition of TfOH and reverts to its original ratio after subsequent addition of a base. Concomitantly, the band at 1075 cm^–1^ shifts to approximately 1071 cm^–1^ after addition of triflic acid and back to 1075 cm^–1^ after addition of a base. The same happens to the band at 1575 cm^–1^, but to a lesser extent, exhibiting a reversible shift of ca. 2 cm^–1^.

It should be noted that although triethylamine (Et_3_N) has a Raman band at 1000 cm^–1^, it has more intense bands at 1068 and 1452 cm^–1^ (Figure S7), neither of which are observed in the SERS spectrum (Figure 2 (right, blue)) and hence the contribution of Et_3_N to the intensity observed at 1000 cm^–1^ is negligible. Temperini et al. noted pH-induced changes in the SERS spectra of SAMs of thionicotinamide and thioisonicotinamide on gold and attributed these to changes in the protonation state of the (non-bound) amino group.^45^ The pH change required to induce changes in the SERS spectrum of thiophenol (p*K*_a_ (aq.) of 6.5–8.0)^11^ is substantial, as demonstrated by the relative invariance of the spectrum obtained on aggregated gold colloid in neutral, basic (pH 13), and acidic (pH 0) water; hence, substantial changes are observed only in aprotic solvents with strong acids such as triflic acid.

**Figure 3:**
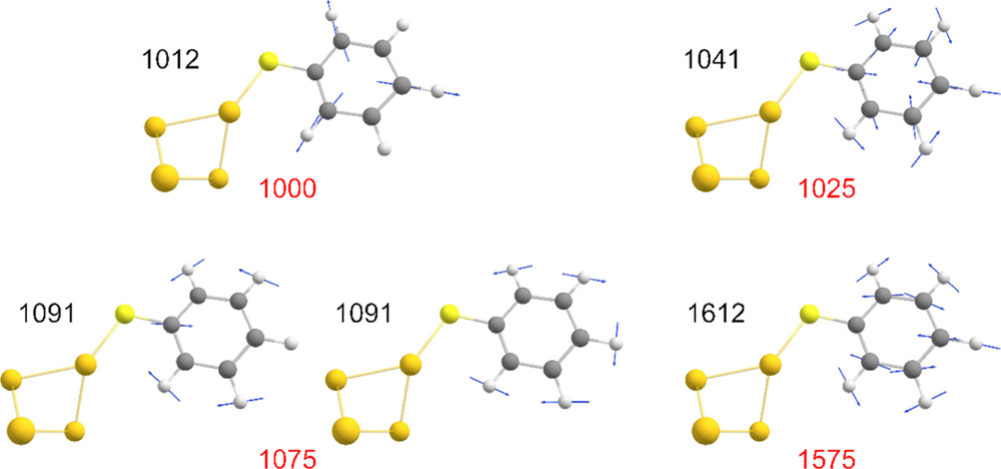
Characteristic vibrational modes of computational model **PhS-Au**_**4**_ (black) and the corresponding experimental SERS bands of **PhS-Au** SAMs (red). The calculated values (in cm^–1^) are of Raman bands after Gaussian broadening, which produces a single band for the frequencies at 1090.98 and 1094.8 cm^–1^. A scaling factor was not applied to the calculated frequencies.

The Raman bands of interest are assigned to their respective vibrational modes by comparison with the calculated Raman spectra of protonated and deprotonated thiophenolato Au_4_ clusters (Figures 3, S10 and Table S2). Earlier studies by Li et al., using a monometallic complex as a computational model,^19^ led to the assignment of the band at 1000 cm^–1^ to ring out-of-plane deformation and C–H out-of-plane bending, the band at 1025 cm^–1^ to ring in-plane deformation and C–C symmetric stretching, the band at 1075 cm^–1^ to C–C symmetric stretching and C–S stretching, and the band at 1575 cm^–1^ to C–C symmetric stretching. Indeed, our assignments are in qualitative agreement, with the band at 1000 cm^–1^ corresponding to a ring breathing mode and the other three bands primarily involving C–H wagging, with the inclusion of a C–S stretch for 1075 cm^–1^ and a C–C stretch for 1575 cm^–1^.

Li et al. conclude from the similarities between the SERS spectrum of **PhSH** on Au colloid and the calculated Raman spectrum of their monomeric computational model and the lack of predicted S–H stretching and CSH bending modes (at 2571 and 901 cm^–1^, respectively) that the adsorbed species on gold colloid is indeed deprotonated. A band at 933 cm^–1^ assigned by Holze as due to a CSH bending mode of **PhSH-Au** under acidic conditions is due to the Cl=O stretching mode of the ClO_4_^–^ ion (see the Supporting Information for details).^27^ It should be noted that Wan et al. also did not observe the S–H stretch in the FTIR spectrum of thiophenol adsorbed onto Au(111).^46^ Since the SERS spectra of **PhS-Au** SAMs reported here do not show characteristic bands for a protonated sulfur atom either, even in acidic media (Figures S4, S8, and S9), we draw the same conclusion that the adsorbed thiophenol molecules are deprotonated.

Our calculations predict a similar change in relative intensities of the Raman bands at 1000 and 1025 cm^–1^ upon addition of a proton to the system, that is, the ratio 1000/1025 cm^–1^ decreases upon going from **PhS-Au**_**4**_ to **PhSH-Au**_**4**_ (Figure S11), as is observed experimentally upon acidification of the medium, in which a **PhS-Au** SAM is immersed. On the other hand, the calculated Raman bands of the neutral deprotonated thiophenolato gold cluster (**PhS-Au**_**4**_), associated with the experimental bands at 1075 and 1575 cm^–1^, shift to higher wavenumber for the cationic protonated system (**PhSH-Au**_**4**_, Figure S11 and Table S2), while the opposite is observed experimentally for **PhS-Au** SAMs in acidic media (vide supra).

This inconsistency between theory and experiment, combined with the change of the Au_4_ cluster from tetrahedral to a flat diamond-like geometry upon addition of a proton to the structure (Figure S10), demonstrates that this computational model is insufficient for obtaining accurate calculated Raman spectra of a protonated, and therefore positively charged, thiophenolato-gold system. Tetsassi Feugmo and Liégeois also calculated the vibrational spectra of adsorbed thiophenol on multi-atomic gold clusters and showed good general agreement with experimental spectra, albeit less good in the region of interest around 1000 cm^–1^ (i.e., the bands at 1000 and 1025 cm^–1^).^47^ Notably, the authors showed that a change in the orientation of the thiophenolato moiety, with respect to the gold atoms, produced changes in Raman band intensities, and indeed, the optimized geometry of **PhSH-Au**_**4**_ in the present study exhibits a smaller C–S–Au angle than in **PhS-Au**_**4**_. While the experimentally observed changes in relative intensities can be reproduced to a certain extent by computational models, it is clear that the employed gold clusters are inadequate representations of a bulk gold surface, in particular, when introducing a positive charge to the system, for example, a proton. Hence, while the changes observed could be correlated to changes in orientation of the PhS-unit, it is unclear whether protonation or rather a change in local pH or local electric field stimulates this change.

An alternative approach to describing a gold surface, in which introduced charges can be delocalized, is to make use of periodicity in a computational model. Zayak et al. achieved quantitatively accurate results in this way, for calculated Raman spectra of thiophenol on planar Au(111) surfaces in comparison with experimental SERS spectra.^18^ Such a periodical computational method seems promising for obtaining meaningful values for the Raman shifts and intensities of adsorbed thiophenol in both the deprotonated and protonated states. It should be emphasized that, for any comparison between computed and experimental intensities, there will be differences due to the dependence of the experimental values on the experimental aspects (e.g., angle of incidence and polarization of beams), whereas the simulations are an average of these parameters.^47^

### Raman Spectroelectrochemistry

**Figure 4:**
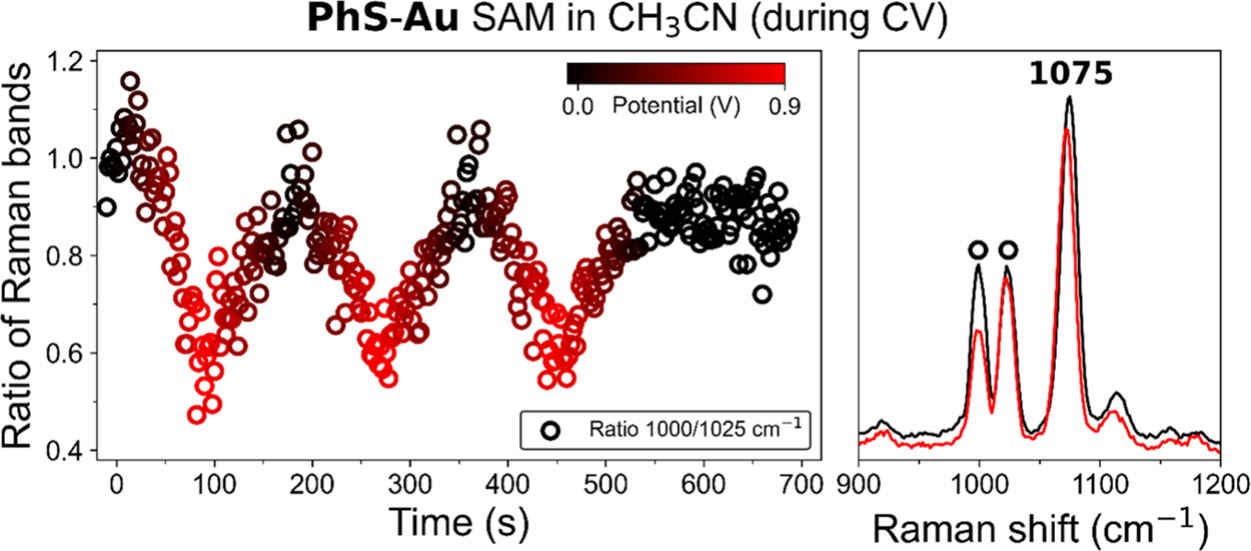
(Left) Changes in ratio of intensities of the Raman bands at 1000 and 1025 cm^–1^ (circle) during cyclic voltammetry of **PhS-Au** in CH_3_CN with 0.1 M TBAPF_6_. Potential is indicated by color from 0.0 V (black) to 0.9 V (red) versus Ag/AgCl. (Right) Corresponding SERS spectra (λ_exc_ 785 nm) at 0.0 V (black) and 0.9 V (red).

**Figure 5:**
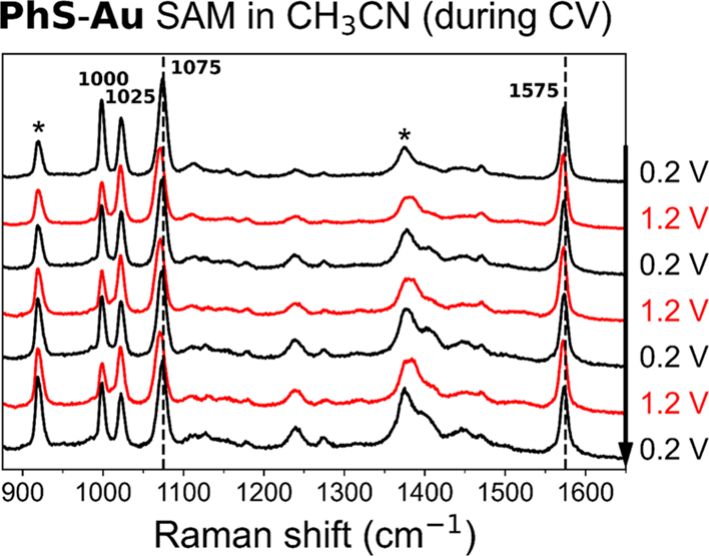
SERS spectra (λ_exc_ 785 nm) of **PhS-Au** at 0.2 V (black) and 1.2 V (red) versus Ag/AgCl during cyclic voltammetry (three cycles, top to bottom) in CH_3_CN with 0.1 M TBAPF_6_. Spectra are normalized on the band at 1575 cm^–1^ and offset for a better comparison. Asterisk denotes CH_3_CN Raman bands.

Although Wan et al. reported the desorption of **PhS-Au** at potentials more positive than 1 V,^46^ destructive oxidation of the underlying Au surface to AuCl_4_, due to the presence of chloride in the perchlorate electrolyte used in that study, is likely to catalyze this process. In our present studies, using chloride-free electrolytes, such desorption was not observed, with only minor but significant changes overall in the SERS spectrum during cyclic voltammetry in acetonitrile up to 0.9 or 1.2 V (Figures 4, 5 and S12) and in KClO_4_ (aq) or HClO_4_ (aq) (Figures S12–S14).

Specific changes to relative band intensities show close correspondence to changes to the SERS and Raman spectrum observed upon addition of an acid or base to the electrolyte (Figure 2, vide supra). In particular, the relative intensity of the bands at 1000 and 1025 cm^–1^ shows a small but reproducible change as the potential reaches 0.9 V, with the band at 1025 cm^–1^ gaining intensity relative to the band at 1000 cm^–1^. The original ratio of intensities is recovered after the potential has returned to 0.0 V (Figure 4).

**Figure 6:**
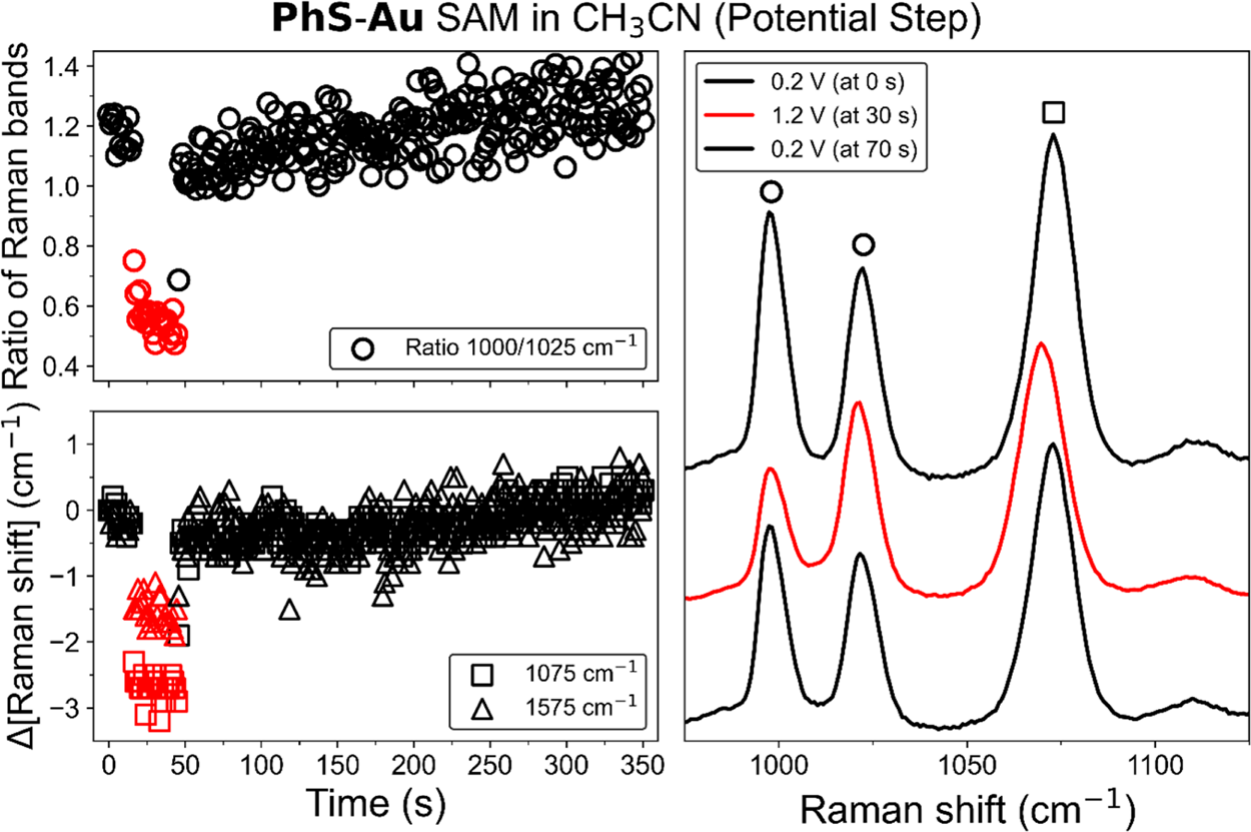
(Left) Changes in the ratio of intensities of the Raman bands at 1000 and 1025 cm^–1^ (circle) and the changes in Raman shift of the bands at 1075 cm^–1^ (square) and 1575 cm^–1^ (triangle) of **PhS-Au** in CH_3_CN with 0.1 M TBAPF_6_ during a potential step experiment between 0.2 V (black) and 1.2 V (red) versus Ag/AgCl. (Right) Corresponding SERS spectra (λ_exc_ 785 nm) of **PhS-Au** at 0.2, 1.2, and at 0.2 V again (top to bottom).

Noticeable secondary, but slight, changes to the SERS spectrum at different potentials are the few-wavenumber shifts to lower frequencies of the Raman bands at 1075 and 1575 cm^–1^, both in CH_3_CN (Figures 5 and 6) and in aqueous media (Figures S13–S15).

A potential step experiment exhibits more rapid spectral changes (Figure 6), ∼3 s versus ∼100 s during cyclic voltammetry, which are a consequence of the equilibria involved and mass transport by diffusion. During cyclic voltammetry, the gradual change in potential results in a likewise gradual change in proton concentration at the surface, while the rate of proton diffusion away from the gold surface is the same as during potential step experiments, resulting in a continuously changing SERS spectrum. In contrast, applying a sudden change in potential from the open circuit potential to one in which water oxidation can proceed rapidly lowers the pH at the electrode as the Nernst diffusion layer is not fully established in this case.

The observation of identical changes in the SERS spectrum of a **PhS-Au** SAM both during cyclic voltammetry and during acid–base cycling is consistent with changes in pH at the electrode, possibly inducing variations in molecular orientation proposed earlier.^25,28,47^ Indeed, oxidation of adventitious water at the roughened gold electrode (at >0.8 V vs Ag/AgCl) will result in an increase in proton concentration (i.e., lower pH) at the surface. The change in ratio of the bands at 1000 and 1025 cm^–1^, as well as the shifts in frequency of the bands at 1075 and 1575 cm^–1^, are a direct manifestation of the local pH change and are therefore useful indicators of local pH. During the return cycle to lower potentials, although protons are no longer generated, the rate of increase of pH at the electrode surface toward that of the bulk solution is limited by diffusion of solvents and general acid present. Hence, although the SERS spectrum reverts to its initial state as the potential becomes less positive and, therefore, the electrochemically induced spectral changes are overall reversible, the rate of change is diffusion-limited.

## Conclusions

In this contribution, we show that the SERS spectrum of thiophenol on gold is sensitive to the local pH, that is, the pH at the surface, manifested in subtle changes to the relative ratio and position of certain bands. We show that several band assignments made in earlier studies need to be reconsidered. In particular, the assignment of variations in the SERS spectra of thiophenol to orientation with respect to the surface should also consider the driving force that induces this change. Beyond specific assignments, the changes observed during cyclic voltammetry together with studies of acid–base cycling of the SAMs on roughened gold bead electrodes show clearly the relation between positive applied potentials (where oxidation of water can occur) and transient decreases in pH that match those achieved by addition of strong acids such as triflic acid.

Furthermore, our calculated Raman spectra of a tetrahedral gold cluster complexed with a thiophenolato moiety show changes upon introduction of a proton to the system. The experimentally observed spectral changes could be due to changes in orientation of the thiophenol moiety with respect to the surface, as reported before, or can be driven by protonation and that this computational model does not describe self-assembled monolayers of thiols on gold surfaces when extra charges, that is, protons, are introduced. Further computational work incorporating periodicity is expected to improve the accuracy in predicting (changes in) Raman intensities, which were not readily explained by the model we apply.

## Supporting Information Available

Details of synthesis and characterization, spectroscopic and electrochemical data, and computational details (PDF)
