# Supplementary material for: pH-Induced Changes in the SERS Spectrum of Thiophenol at Gold Electrodes during Cyclic Voltammetry
Source: J Phys Chem C Nanomater Interfaces. 2022 Apr 20;126(17):7680–7. doi: 10.1021/acs.jpcc.2c00416 (PMC9082592; doi:10.1021/acs.jpcc.2c00416)
Supplement: Supplementary file 1 — jp2c00416_si_001.pdf [file jp2c00416_si_001.pdf]

Online Supporting Information

pH-Induced Changes in the SERS Spectrum of Thiophenol at Gold Electrodes during Cyclic Voltammetry

Jorn D. Steen, Anouk Volker, Daniël R. Duijnstee, Andy S. Sardjan, Wesley R. Browne*

Molecular Inorganic Chemistry, Stratingh Institute for Chemistry, Faculty of Science and Engineering, University of Groningen, Nijenborgh 4, 9747AG, Groningen, The Netherlands.

*email: w.r.browne@rug.nl

TABLE OF CONTENTS

Synthetic procedures.......... 2

Gold thiophenolate.......... 2

Copper thiophenolate.......... 2

Supporting Experimental Data.......... 2

Computational Details.......... 12

Frequency calculations.......... 12

Cartesian coordinates.......... 15

References.......... 17

SYNTHETIC PROCEDURES

Gold thiophenolate

The **AuSPh** complex was synthesized according to the procedure reported by Dyadchenko et al. using thiophenol and tetrachloroauric acid.[1,2]

A solution of thiophenol (99 mg, 0.9 mmol) in methanol (2 mL) was added slowly, over a few minutes, to a stirred solution of $HAuCl_4 \cdot xH_2O$ (65 mg, 0.19 mmol) in a mixture of water (1 mL) and methanol (4 mL), during which a white precipitate formed. The reaction mixture was stirred for 1 h, after which the white, milky suspension was filtered over a paper filter. The light-yellow residue was washed with methanol (5 mL), acetone (5 mL), and diethyl ether (5 mL), and dried in air overnight, yielding 39 mg (0.13 mmol, 70%) of **AuSPh** as a yellow powder.

Copper thiophenolate

The **CuSPh** complex was synthesized according to the procedure reported by Carron and Hurley using thiophenol and copper(II) chloride.[3]

Thiophenol (neat, 1.78 g, 16 mmol) was added to a solution of $CuCl_2$ (203 mg, 1.5 mmol) in H_2O (10 ml), upon which immediately yellow precipitate formed. The reaction mixture was stirred for 1.5 h and subsequently filtered over a paper filter. The yellow residue was washed with methanol (30 ml) and then dried in air overnight, yielding 7 mg (0.04 mmol, 3%) of **CuSPh** as a yellow powder.

The solid state Raman spectrum (λ_{exc} 785 nm) of the product **CuSPh** is in agreement with that reported by Carron and Hurley,[3] but shows a residual small impurity of diphenyl disulfide as evidenced by the S-S stretch at 542 cm^{-1} (Figure S1).[4,5]

SUPPORTING EXPERIMENTAL DATA

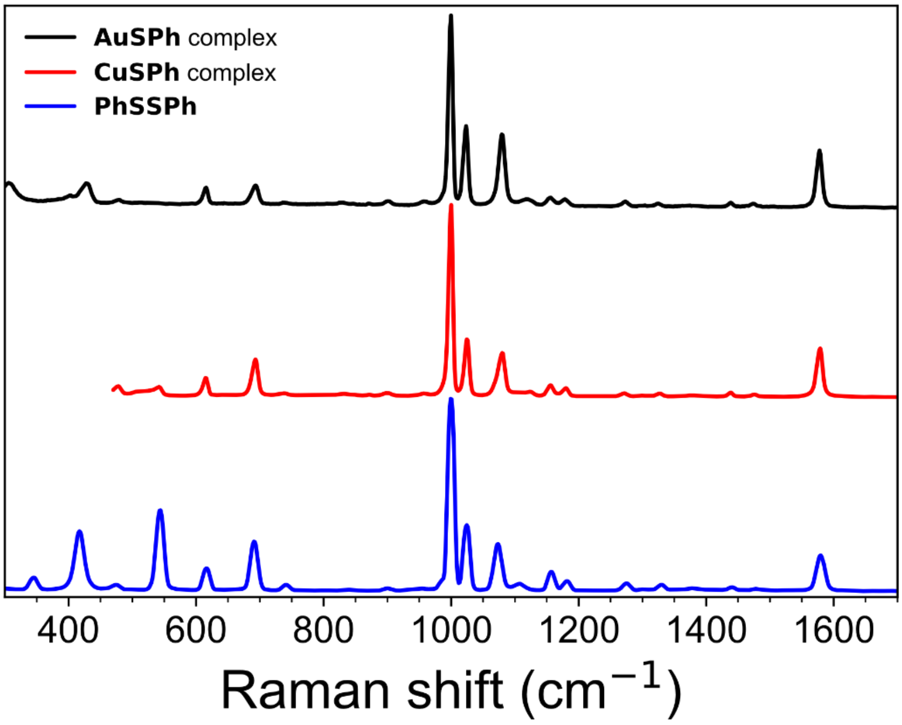

*Figure S1 Solid-state Raman spectra (λ_{exc} 785 nm) of **AuSPh** (black), **CuSPh** (red), and **PhSSPh** (blue). The spectra are normalized and offset for clarity.*

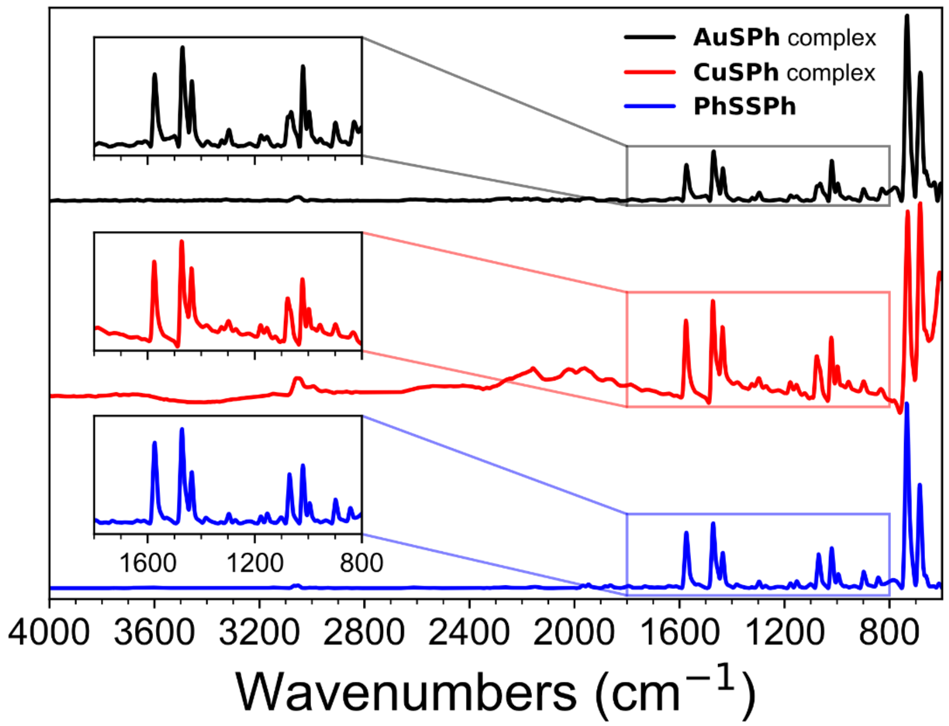

*Figure S2 ATR-FTIR spectra of **AuSPh** (black), **CuSPh** (red), and **PhSSPh** (blue). The spectra are normalized and offset for clarity.*

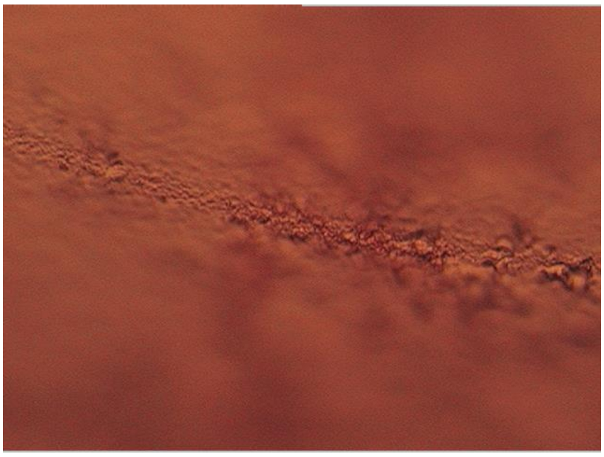

Figure S3 Microscope image (50x) of the electrochemically roughened surface of a gold bead.

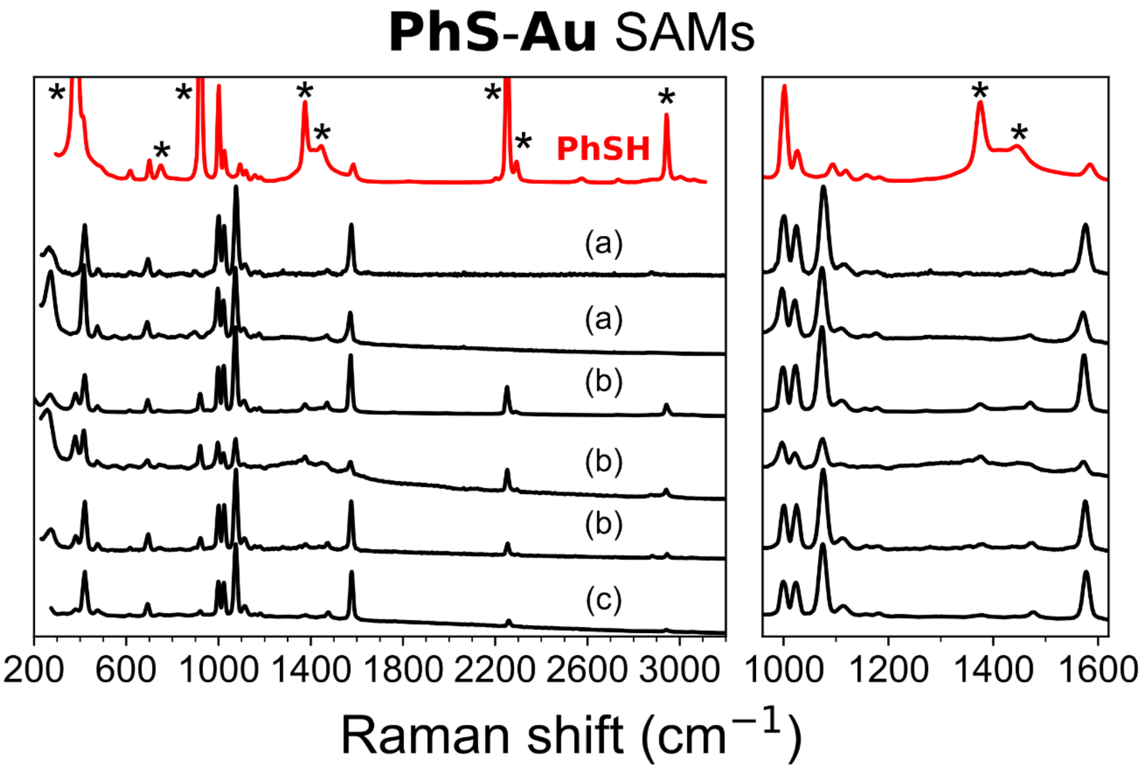

*Figure S4 Raman spectrum (λ_{exc} 785 nm) of 0.5 M **PhSH** in CH_3CN (red), and SERS spectra (λ_{exc} 785 nm) of **PhS-Au** SAMs on roughened gold beads (black), (a) under dry conditions (no solvent), (b) in CH_3CN, and (c) in CH_3CN with 0.1 M $TBAPF_6$. The spectra are normalized and offset for clarity. Asterisk denotes CH_3CN solvent bands.*

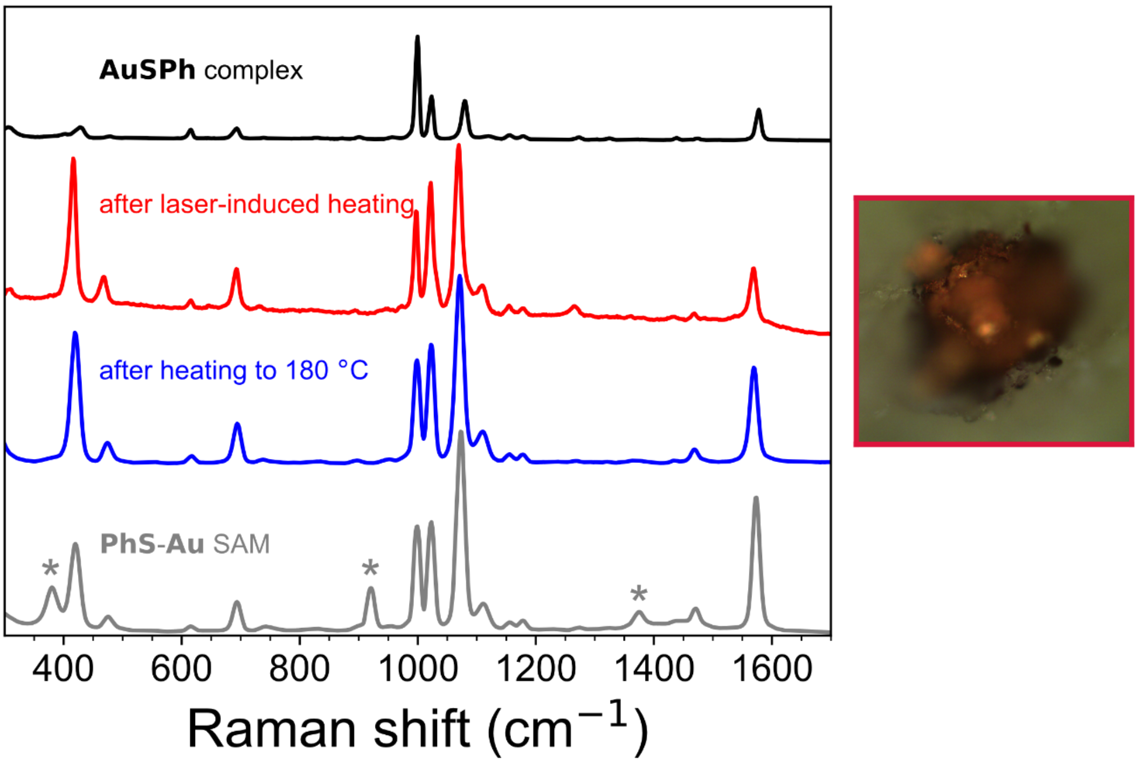

*Figure S5 (left) Solid state Raman spectra (λ_{exc} 785 nm) of the **AuSPh complex** before (black) and after laser-induced heating (red) and thermal heating to 180 °C in a microscope heating stage (blue), compared to the SERS spectrum (λ_{exc} 785 nm) of a **PhS-Au** SAM on a roughened gold bead in CH_3CN (gray). The spectra are normalized*

and offset for clarity. Asterisk denotes CH_3CN solvent bands. (right) Microscope image (50x) of the ***AuSPh*** *sample after laser-induced heating.*

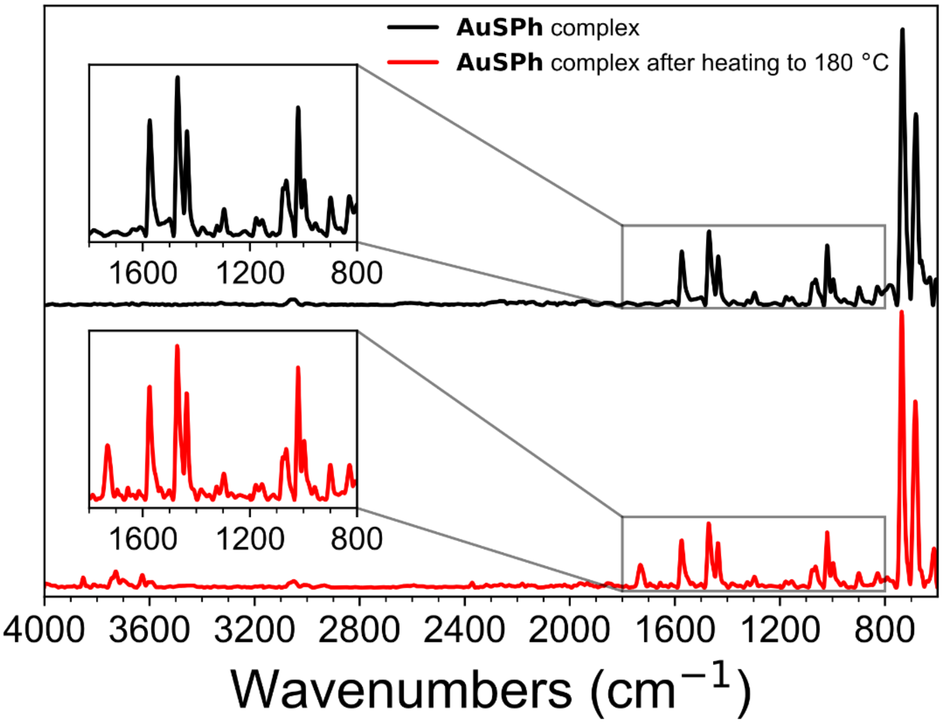

Figure S6 ATR-FTIR spectra of the ***AuSPh complex*** *before (black) and after (red) heating to 180 °C in a microscope heating stage. The spectra are normalized and offset for clarity.*

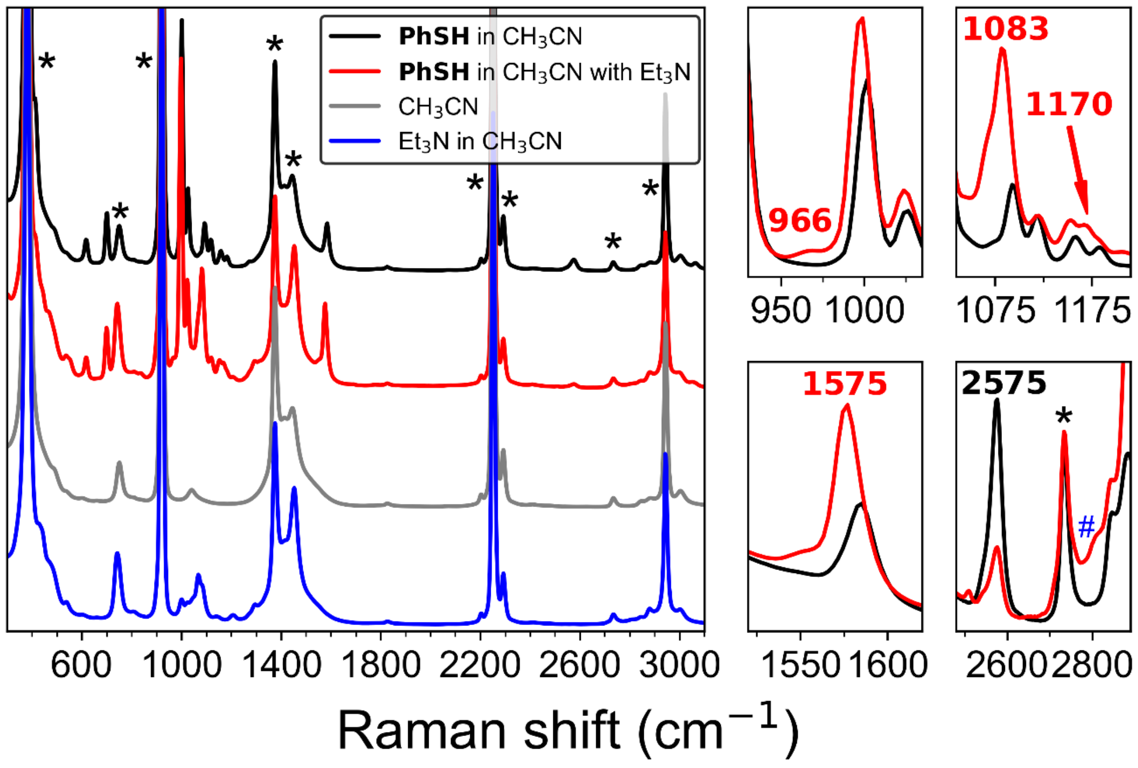

Figure S7 Raman spectra (λ_{exc} 785 nm) of 0.5 M ***PhSH*** *in CH_3CN before (black) and after (red) addition of Et_3N. Spectra of CH_3CN (grey) and Et_3N in CH_3CN (blue) are added for reference. The spectra are normalized and offset for clarity. Asterisk denotes CH_3CN bands. Hash denotes Et_3N bands.*

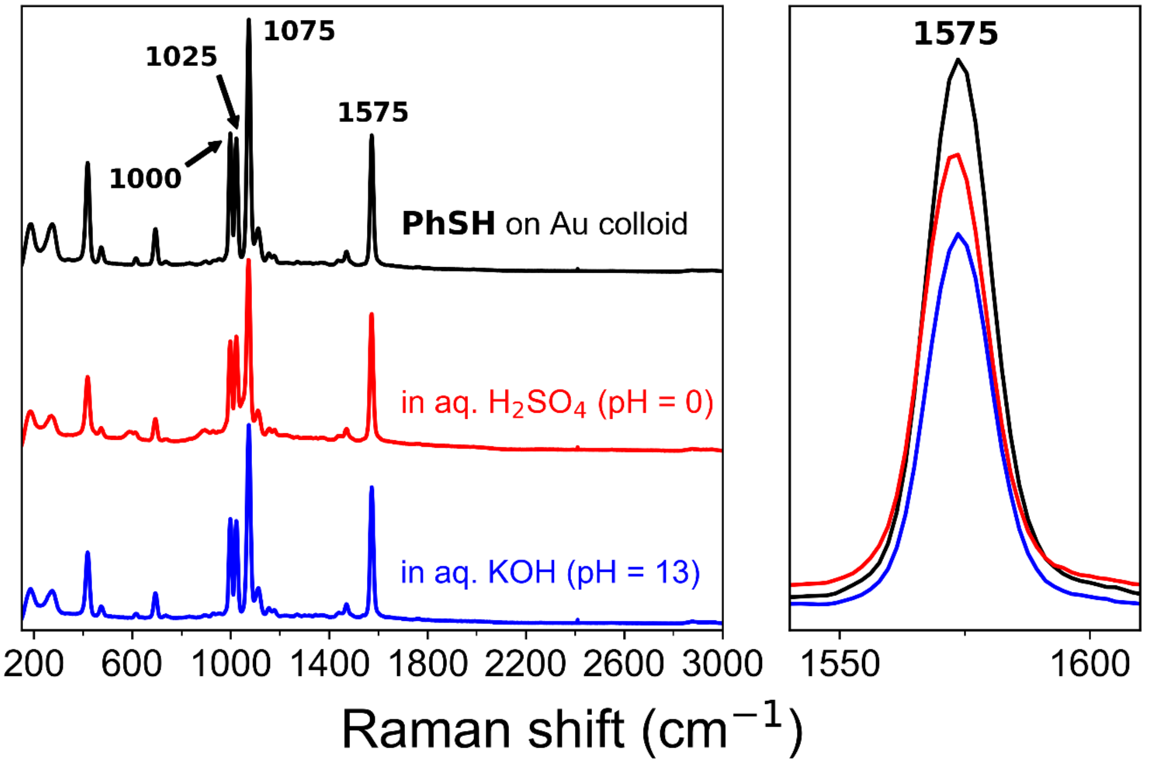

*Figure S8 SERS spectra (λ_{exc} 785 nm) of **PhSH** on aggregated gold colloid in H_2O (black) at pH = 0 (red) and pH = 13 (blue). The spectra are normalized and offset for clarity.*

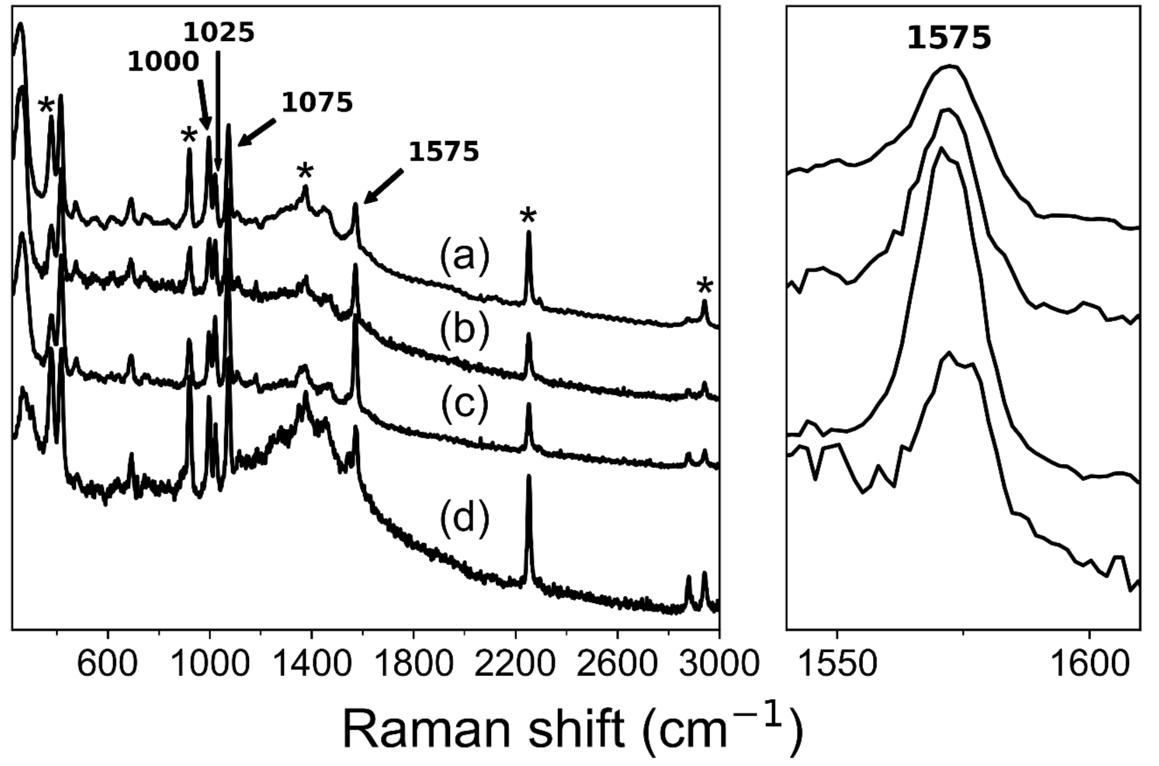

*Figure S9 SERS spectra (λ_{exc} 785 nm) of **PhS-Au** on a roughened gold bead in CH_3CN (a) before, (b) immediately after addition of TfOH, (c) after waiting several minutes, and (d) after subsequent addition of Et_3N. The spectra are normalized and offset for clarity. Asterisk denotes CH_3CN bands.*

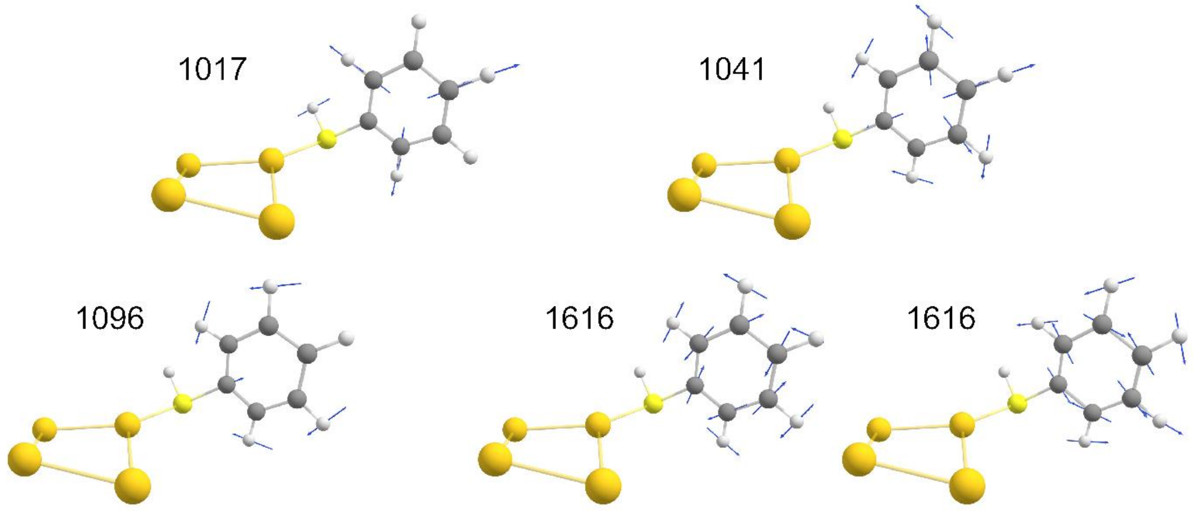

*Figure S10 Characteristic vibrational modes of computational model **PhSH-Au₄**. The values (in cm^{-1}) are of the Raman bands after Gaussian broadening, which produces a single band for the frequencies at 1615.73 and 1617.24 cm^{-1}. A scaling factor was not applied to the calculated frequencies. See Table S2 for comparison with deprotonated and experimental Raman bands.*

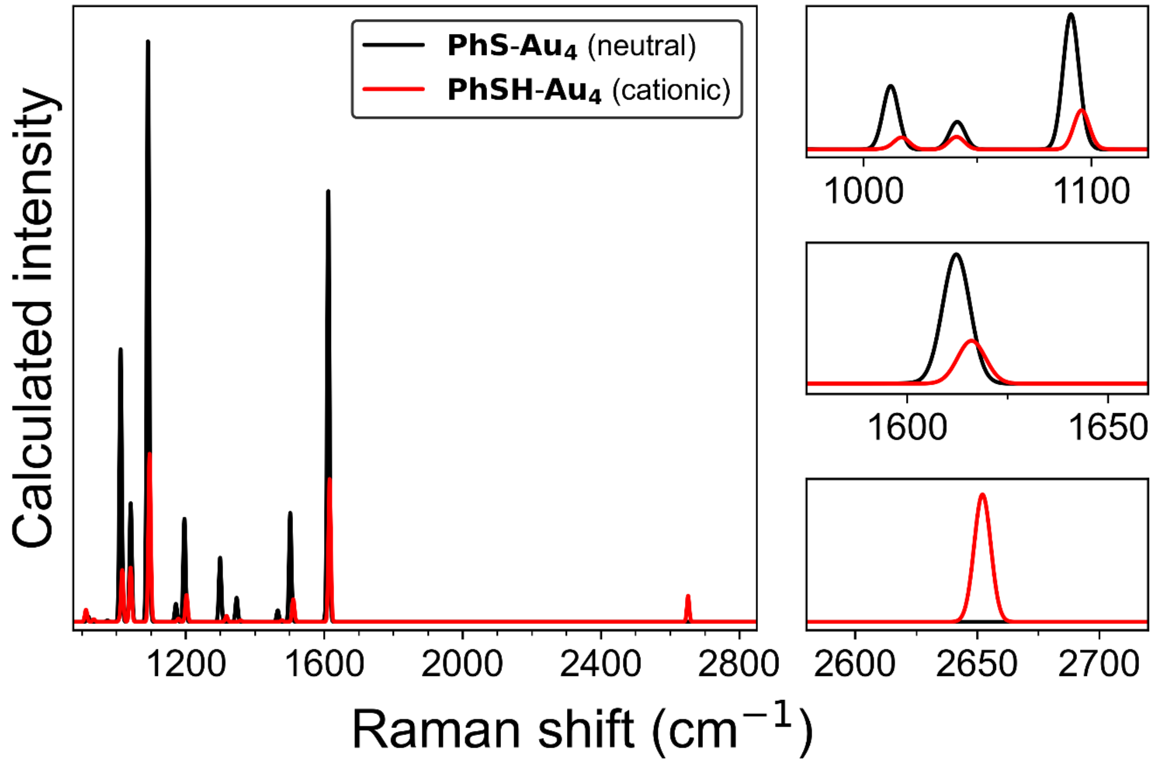

*Figure S11 Calculated Raman spectra of the neutral deprotonated (**PhS-Au₄**, black) and the cationic protonated (**PhSH-Au₄**, red) thiophenolato gold clusters. The Raman intensities were calculated from the obtained Raman activities (see Computational Details), and a Gaussian broadening with a full width at half maximum of 8 cm^{-1} was applied to the Raman frequencies.*

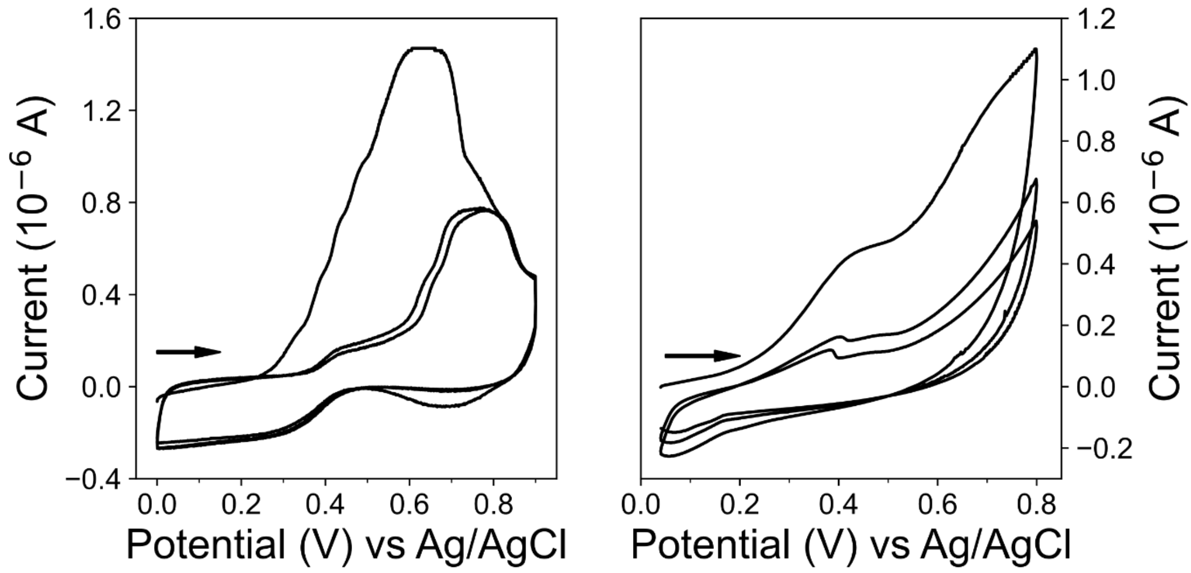

*Figure S12 Cyclic voltammograms of **PhS-Au** in (left) CH_3CN (0.1 M $TBAPF_6$) and (right) 0.5 M $KClO_4$ (aq). Arrows indicate starting point and initial direction.*

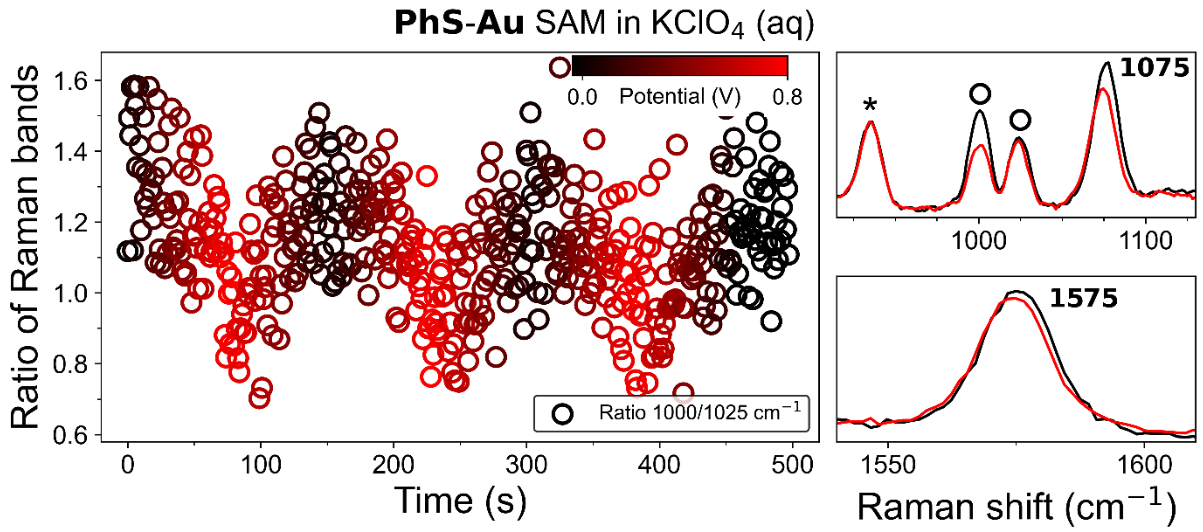

*Figure S13 (left) Changes in ratio of the Raman bands at 1000 cm^{-1} and 1025 cm^{-1} (circle) during cyclic voltammetry of **PhS-Au** in 0.5 M $KClO_4$ (aq). Potential is indicated by color from 0.0 V (black) to 0.8 V (red) vs Ag/AgCl. (right) Corresponding SERS spectra (λ_{exc} 785 nm) at 0.0 V (black) and 0.8 V (red). The spectra were normalized on the perchlorate band denoted by the asterisk.*

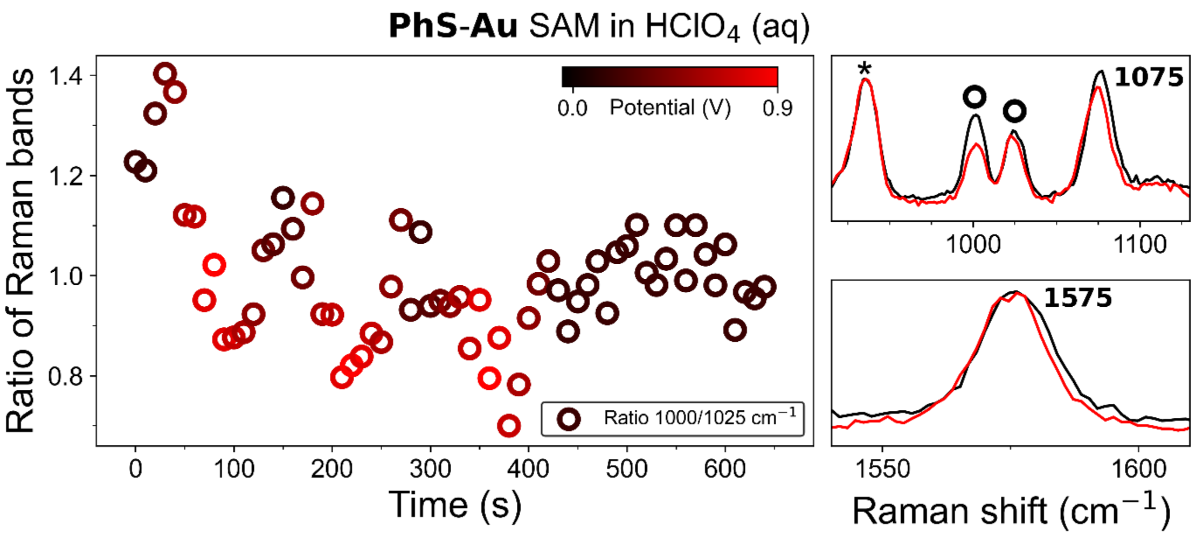

*Figure S14 (left) Changes in ratio of the Raman bands at 1000 cm^{-1} and 1025 cm^{-1} (circle) during cyclic voltammetry of **PhS-Au** in 0.5 M $HClO_4$ (aq). Potential is indicated by color from 0.0 V (black) to 0.9 V (red) vs Ag/AgCl. (right) Corresponding SERS spectra (λ_{exc} 785 nm) at 0.0 V (black) and 0.9 V (red). The spectra were normalized on the perchlorate band denoted by the asterisk.*

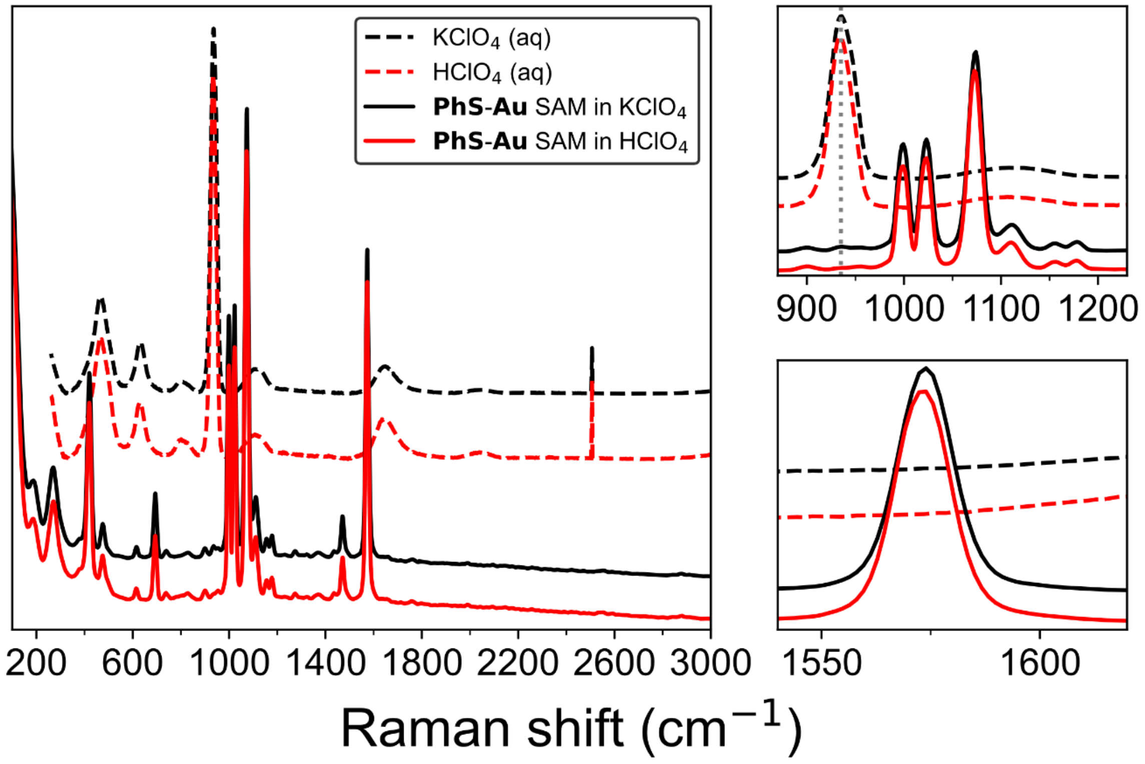

*Figure S15 SERS spectra (λ_{exc} 785 nm) of **PhS-Au** (SAM on a roughened gold bead) in aqueous solutions of $KClO_4$ (black, solid) and $HClO_4$ (red, solid), and Raman spectra (λ_{exc} 785 nm) of 0.5 M $KClO_4$ (aq) (black, dashed) and 0.5 M $HClO_4$ (aq) (red, dashed). The spectra are normalized and offset for clarity. The dashed gray line indicates the Cl=O stretching mode of the perchlorate ion at 933 cm^{-1}. The spike at 2550 cm^{-1} in the spectra of $KClO_4$ (aq) and $HClO_4$ (aq) comes from a hot pixel artefact.*

Holze reported earlier an additional band at 933 cm^{-1} during cyclic voltammetry in water with perchlorate as electrolyte, which was assigned to the CSH bending mode of **PhSH-Au**.[6] This band was assigned earlier by Scott et al.[7] at 916 cm^{-1} for neat thiophenol (**PhSH**) which prompted us to examine the reported band further. In sulfuric acid, the same SERS spectrum was obtained except for the band at 933 cm^{-1} (Figure S8). This band corresponds to the Cl=O stretching mode of the ClO_4^- ion rather than a mode of thiophenolate (Figure S15). The apparent increase with lowering of pH (using $HClO_4$ (aq)) noted by Holze is most likely due to a change in overall SERS intensity relative to the Raman scattering of the ClO_4^- ion in solution.

*Table S1 Raman shifts (cm^{-1}) and vibrational mode assignments for **PhSH**, **PhS-Au** SAMs, and the **AuSPh** complex under various experimental conditions. [a]See Figure 3. oop = out-of-plane, ip = in-plane, sym = symmetric.*

**Vibrational mode**	**PhSH**	**PhSH with base (deprotonation)**	**PhS-Au SAM**	**PhS-Au SAM at low pH**	**AuSPh complex**	
					Before heating	*After heating*
Au-S stretching[6,8]	-	-	270 - 275	265 - 270	305	280
C-S-H bending[7,8]	under CH_3CN band; 916[7]	under CH_3CN band	-	902(calc)[8]	-	-
ring *oop* deformation & C-H *oop* bending[6,8]	1002	998	1000[a]	1000	998	998
ring *ip* deformation & C-C *sym* stretching[6,8]	1026	1024	1025[a]	1025	1021	1021
C-C *sym* stretching and C-S stretching[8]	1095	1083	1075[a]	1071	1080	1070
C-C *sym* stretching[8]	1585	1575	1575[a]	1573	1575	1570
S-H stretching[6–9]	2575	2575 (lower intensity)	-	2571(calc)[8]	-	-

*Table S2 Comparison of characteristic experimentally observed Raman bands of **PhS-Au** SAMs at 1000, 1025, 1075, and 1575 cm^{-1}, in neutral and acidic media, with the corresponding assigned bands for the neutral deprotonated **PhS-Au$_4$** and cationic protonated **PhSH-Au$_4$** computational models, respectively. A scaling factor was not applied to the calculated frequencies. Note that, as a result of Gaussian broadening, the calculated Raman band of **PhS-Au$_4$** at 1091 cm^{-1} originates from the frequencies 1090.98 and 1094.8 cm^{-1} (Figure 3), and that of **PhSH-Au$_4$** at 1616 cm^{-1} from the frequencies 1615.73 and 1617.24 cm^{-1} (Figure S10).*

		1000 cm^{-1}		*1025 cm^{-1}*		*1075 cm^{-1}*		*1575 cm^{-1}*	
Exp.	Calc.	Exp.	Calc.	Exp.	Calc.	Exp.	Calc.	Exp.	Calc.
**PhS-Au** (neutral)	**PhS-Au$_4$**	1000	1012	1025	1041	1075	1091	1575	1612
**PhS-Au** (acidic)	**PhSH-Au$_4$**	1000	1017	1025	1041	1071	1096	1573	1616
Shift		0	5	0	0	-4	5	-2	4

COMPUTATIONAL DETAILS

Frequency calculations

Raman intensities (I_i) were calculated from the Raman activities (S_i) using the following equation:[10,11]

$$\mathrm{I}_i = \frac{f\,(\nu_0 - \nu_i)^4\, S_i}{\nu_i \left(1 - e^{\frac{-hc\nu_i}{k_B T}}\right)}$$

where f is a normalization factor, ν_0 is the laser wavenumber, ν_i is the ith vibrational mode, h is Planck's constant, c is the speed of light (in cm s^{-1}), k_B is Boltzmann's constant, and T is the temperature.

*Table S3 Calculated Raman frequencies of **PhS-Au4** (deprotonated, neutral thiophenolato Au4 cluster) and the corresponding Raman activities and intensities ($f = 10^{-12}$, $\nu_0 = 12739$ cm^{-1}, $T = 293$ K).*

Frequency (cm^{-1})	Raman activity	Raman intensity
11.37	11.012326	468047.4802
19.3	130.573465	1958569.829
20.75	183.775735	2392067.742
27.2	31.172602	239341.6375
50.22	395.521253	934156.8725
57.09	47.683377	88371.80168
75.29	50.651077	55981.34557
79.62	165.845507	165317.8077
86.85	2497.401816	2122278.07
99.34	287.010625	191026.8168
153.76	404.84374	124625.8111
177.17	26.192523	6335.610641
216.7	63.26941	10961.09807
384.66	1046.700719	74686.11376
411.82	1.664643	107.5791747
428.96	362.110836	22070.23314
483.51	15.937113	819.8960596
630.3	31.709951	1132.812989
700.21	132.213611	4097.851102
705.46	7.371597	226.1898762
755.52	11.30533	316.3239074
849.98	18.775014	448.2217974
918.13	19.031642	409.2420319
973.91	5.176608	102.6976331
988.87	0.125572	2.439593774
1012.03	1102.052877	20738.60225
1041.22	497.874106	9007.671661
1090.98	2496.311997	42319.69485
1094.8	197.396615	3330.100478
1171.79	88.534799	1356.930257

1196.46	526.298825	7829.98739
1299.55	368.681436	4866.476148
1347.35	145.626316	1822.584555
1466.2	78.645361	866.8252116
1502.29	779.807157	8280.600964
1601.05	13.358472	128.4518113
1612.3	3443.156304	32744.22333
3161.09	23.33997	62.13646163
3165.25	404.512377	1073.623983
3175.52	262.505063	691.4913011
3178.73	80.475808	211.4912521
3189.49	658.03683	1715.747507

Table S4 Calculated Raman frequencies of ***PhSH-Au₄*** *(protonated, cationic thiophenolato Au_4 cluster) and the corresponding Raman activities and intensities ($f = 10^{-12}$, $v_0 = 12739$ cm^{-1}, $T = 293$ K).*

Frequency (cm^{-1})	Raman activity	Raman intensity
18.81	11.236936	177264.4487
28.09	0.819075	5907.573271
41.59	12.223145	41355.46151
60.05	5.397336	9095.359697
65.02	38.302088	55608.68151
81.14	13.731338	13219.31536
89.82	6.592052	5268.158996
139.57	34.046286	12391.3982
141.08	81.272742	29031.34656
175.82	35.278114	8643.962468
263.65	11.290331	1428.725811
284.25	25.18823	2834.047571
404.08	39.959421	2654.18308
409.94	19.416828	1263.142411
480.55	8.463327	439.167078
569.63	34.887264	1430.450192
627.46	11.36297	408.4251361
701.3	16.240033	502.2930528
703.9	34.578638	1064.177318
759.14	3.70959	103.1292754
849.96	3.387276	80.86807338
911.93	42.086574	913.378287
934.57	9.440816	198.1587171
982.07	0.494261	9.693899954
1009.76	9.779614	184.605346
1016.79	209.25447	3912.356949
1040.93	227.716712	4121.503773
1095.78	758.399557	12778.24544

1105.1	7.196228	119.8171269
1179.05	15.391189	233.8267724
1202.55	137.151716	2025.685958
1318.02	34.820672	450.1948618
1358.82	8.767205	108.3541612
1478.33	11.353539	123.5726773
1511.12	161.680984	1701.418818
1615.73	889.461329	8430.315913
1617.24	276.289561	2614.800616
2652.15	505.150307	1971.722295
3175.08	47.443822	125.0169236
3180.96	294.182353	771.8516832
3187.9	234.25258	611.495592
3194.1	52.315696	135.9468336
3199.54	808.410898	2092.376559

Cartesian coordinates

Below are listed the Cartesian XYZ coordinates (Å) of the thiophenolato gold clusters as optimized with DFT.

PhS-Au_4

16

Au	-4.688549000	3.243077000	-1.290265000
Au	-6.134486000	1.202173000	0.051165000
Au	-3.372220000	0.822778000	-1.036107000
Au	-3.866573000	2.218996000	1.164268000
S	-5.209942000	5.066371000	-2.587122000
C	-3.714719000	5.906679000	-3.065440000
C	-2.422750000	5.469945000	-2.754813000
C	-3.866291000	7.086866000	-3.806555000
C	-1.313805000	6.198415000	-3.171787000
H	-2.287278000	4.558671000	-2.186133000
C	-1.469726000	7.371240000	-3.905272000
H	-0.321447000	5.843229000	-2.919645000
C	-2.753196000	7.809103000	-4.220715000
H	-4.859859000	7.439649000	-4.056406000
H	-2.893184000	8.719765000	-4.791189000
H	-0.603325000	7.936024000	-4.226184000

PhSH-Au_4

17

Au	-4.745597000	3.408195000	-1.027260000
Au	-6.895216000	2.219562000	0.247694000
Au	-2.324698000	2.984314000	0.279093000
Au	-4.593361000	2.478932000	1.537269000
S	-4.897924000	4.310595000	-3.217466000
C	-3.573683000	5.510991000	-3.371865000

C	-2.396353000	5.078262000	-3.976849000
C	-3.713546000	6.820253000	-2.919212000
C	-1.341830000	5.972516000	-4.123343000
H	-2.302542000	4.058888000	-4.330159000
C	-1.469352000	7.285047000	-3.679285000
H	-0.423544000	5.640689000	-4.591165000
C	-2.655259000	7.706359000	-3.083828000
H	-4.630559000	7.144855000	-2.444624000
H	-2.758884000	8.727576000	-2.739124000
H	-0.647156000	7.979427000	-3.797733000
H	-5.909554000	5.176399000	-3.016642000

REFERENCES

(1) Dyadchenko, V. P.; Belov, N. M.; Dyadchenko, M. A.; Slovokhotov, Y. L.; Banaru, A. M.; Lemenovskii, D. A. A Complex of Gold(I) Benzenethiolate with Isocyanide: Synthesis and Crystal and Molecular Structures. *Russ. Chem. Bull.* **2010**, *59*, 539–543.

(2) Dyadchenko, V. P.; Belov, N. M.; Lemenovskii, D. A.; Antipin, M. Y.; Lyssenko, K. A.; Bruce, A. E.; Bruce, M. R. M. Synthesis, Crystal and Molecular Structure of Gold(I) Thiophenolate with 4′-Ferrocenyl[1,1′]Biphenylisocyanides. *J. Organomet. Chem.* **2010**, *695*, 304–309.

(3) Carron, K. T.; Hurley, L. G. Axial and Azimuthal Angle Determination with Surface-Enhanced Raman Spectroscopy: Thiophenol on Copper, Silver, and Gold Metal Surfaces. *J. Phys. Chem.* **1991**, *95*, 9979–9984.

(4) Dong, J.; Sheng, Z.; Xue, G. Near-IR Excited Surface Enhanced Raman Scattering from Adsorbed Layer on Nickel by Doping with Silver. *Spectrochim. Acta Part A Mol. Biomol. Spectrosc.* **1995**, *51*, 1031–1041.

(5) Sandroff, C. J.; Herschbach, D. R. Surface-Enhanced Raman Study of Organic Sulfides Adsorbed on Silver: Facile Cleavage of Sulfur-Sulfur and Carbon-Sulfur Bonds. *J. Phys. Chem.* **1982**, *86*, 3277–3279.

(6) Holze, R. The Adsorption of Thiophenol on Gold – a Spectroelectrochemical Study. *Phys. Chem. Chem. Phys.* **2015**, *17*, 21364–21372.

(7) Scott, D. W.; McCullough, J. P.; Hubbard, W. N.; Messerly, J. F.; Hossenlopp, I. A.; Frow, F. R.; Waddington, G. Benzenethiol: Thermodynamic Properties in the Solid, Liquid and Vapor States; Internal Rotation of the Thiol Group. *J. Am. Chem. Soc.* **1956**, *78*, 5463–5468.

(8) Li, S.; Wu, D.; Xu, X.; Gu, R. Theoretical and Experimental Studies on the Adsorption Behavior of Thiophenol on Gold Nanoparticles. *J. Raman Spectrosc.* **2007**, *38*, 1436–1443.

(9) Szafranski, C. A.; Tanner, W.; Laibinis, P. E.; Garrell, R. L. Surface-Enhanced Raman Spectroscopy of Aromatic Thiols and Disulfides on Gold Electrodes. *Langmuir* **1998**, *14*, 3570–3579.

(10) Chaitanya, K.; Santhamma, C.; Prasad, K. V.; Veeraiah, V. Molecular Structure, Vibrational Spectroscopic (FT-IR, FT-Raman), First Order Hyperpolarizability, NBO Analysis, HOMO and LUMO Analysis, Thermodynamic Properties of 3,5-Dimethylbenzophenone by Ab Inito HF and Density Functional Method. *J. At. Mol. Sci.* **2011**, *3*, 1–22.

(11) Cozar, I. B.; Szabó, L.; Leopold, N.; Chiş, V.; Cozar, O.; David, L. IR, Raman, SERS and DFT Study of Pindolol and Verapamil. *J. Mol. Struct.* **2011**, *993*, 308–315.
